# Nurse Registration in Victoria

**Published:** 1919-12-13

**Authors:** 


### Nurse Registration in Victoria.

(By Our Special Correspondent.)

The nursing world in Victoria is just now in a state of ferment over a Bill before our Legislative Assembly "To make provision with respect to the Training Qualifications and Registration of Nurses and for other purposes." *Inter alia*, the Bill provides for the appointment by the Governor in Council of "five members of the Public Service of Victoria as a Board (to be called the Nurses' Board)," such appointees to hold office for a period of three years, and upon expiration thereof to be eligible for reappointment. The powers conferred on this Board, as the Bill stands, include (*a*) "to hold examinations and to appoint examiners, and such examiners shall include persons appointed as representing medical practitioners, nurses registered under this Act, and public hospitals approved by the Board as general training-schools for nurses; (*b*) to decide upon the places where and the times when the examinations are to be held; (*c*) to issue a certificate of registration to any person registered under this Act."

Further than that, powers are conferred on the Board to cancel certificates and suspend nurses. Strong objections have been taken to the Bill as drafted by those interested in the training and treatment of nurses. Among the principal may be cited: (1) The constitution of the Board, and the fact that the claims of nurses for representation on it have been ignored. (2) The powers of a Board consisting solely of civil servants to suspend nurses or cancel their certificates for disobeying regulations not yet in existence, or for misconduct which may mean anything or nothing. (3) That an appeal from the decision of the Board must be to the Minister of Health, whose decision shall be final and without appeal, thus depriving nurses of their undoubted right as individuals to clear their reputations in Courts of Law, which might involve the loss of their livelihood.

The Royal Victorian Trained Nurses' Association has strongly protested against the exclusion of nurses from the proposed Board, and one of its former presidents (Dr. J. W. Springthorpe) has voiced his protest in the daily Press. He points out that the R.V.T.N.A. was founded in 1901, and, in conjunction with its sister association the A.T.N.A. of New South Wales, has ever since managed nursing matters for the whole of Australia. In Victoria it has brought together and combined as training-schools forty-five hospitals, practically all of any size in that State. Its position has been recognised everywhere as almost ideal, and it was the first (outside Great Britain) to obtain the title of "Royal." It has registered 5,000 nurses, and has at present 2,226 on its register. It has sent 845 of its members to the Front, while 153 others have done war work here. The Association is a purely voluntary one, and has no compulsory powers beyond justice and interest. The *Melbourne Age* calls attention to the fact that there is nothing so far in the Bill to prevent a woman practising for gain as a nurse so long as she disclaims being "a registered nurse," and voices its strong objection that those mostly concerned are to have no direct representation on the Board.

